# nNOS–CAPON interaction mediates amyloid‐β‐induced neurotoxicity, especially in the early stages

**DOI:** 10.1111/acel.12754

**Published:** 2018-03-25

**Authors:** Yu Zhang, Zhu Zhu, Hai‐Ying Liang, Lei Zhang, Qi‐Gang Zhou, Huan‐Yu Ni, Chun‐Xia Luo, Dong‐Ya Zhu

**Affiliations:** ^1^ Department of Pharmacology Nanjing Medical University Nanjing China; ^2^ Department of Pharmacy Second Affiliated Hospital of Soochow University Suzhou China

**Keywords:** amyloid‐β, Dexras1, ERK–CREB–BDNF, neurotoxicity, nNOS–CAPON interaction, S‐nitrosylation

## Abstract

In neurons, increased protein–protein interactions between neuronal nitric oxide synthase (nNOS) and its carboxy‐terminal PDZ ligand (CAPON) contribute to excitotoxicity and abnormal dendritic spine development, both of which are involved in the development of Alzheimer's disease. In models of Alzheimer's disease, increased nNOS–CAPON interaction was detected after treatment with amyloid‐β *in vitro,* and a similar change was found in the hippocampus of APP/PS1 mice (a transgenic mouse model of Alzheimer's disease), compared with age‐matched background mice *in vivo*. After blocking the nNOS–CAPON interaction, memory was rescued in 4‐month‐old APP/PS1 mice, and dendritic impairments were ameliorated both *in vivo* and *in vitro*. Furthermore, we demonstrated that S‐nitrosylation of Dexras1 and inhibition of the ERK–CREB–BDNF pathway might be downstream of the nNOS–CAPON interaction.

## 1 INTRODUCTION

Alzheimer's disease is a neurodegenerative disorder characterized by degeneration of specific neurons and is a heavy burden among the aging population. In pathology, abnormal accumulation of amyloid‐β (Aβ), dysfunction of synapses, and hyperphosphorylated tau are typical manifestations of Alzheimer's disease (Scheltens et al., 2016). Multiple mechanisms of Alzheimer's disease have been proposed among which the amyloid cascade hypothesis is the most popular. In the amyloid cascade hypothesis, Aβ triggers damage to neurons and induces disease. However, the toxic mechanism of Aβ is complicated. Several studies have shown that Aβ binds to receptors on neurons and triggers downstream cytotoxic signaling processes (Jarosz‐Griffiths, Noble, Rushworth & Hooper, 2016).

N‐methyl‐D‐aspartate receptor (NMDAR), a classic glutamate receptor in neurons, is a potential Aβ receptor involved in the cytotoxic action of Aβ (Jarosz‐Griffiths et al., 2016). Many reports have shown that Aβ can overactivate extrasynaptic NMDARs on neurons, which causes impaired spines and synapses and neuron death (Zhang, Li, Feng & Wu, 2016). Extrasynaptic NMDARs promote inactivation of ERK, dephosphorylation of CREB, and phosphorylation of p38, while synaptic NMDARs provide a neuroprotective effect by inducing the expression of survival genes and inhibiting the expression of apoptotic genes (Zhang et al., 2016). Memantine, a clinical drug used to treat AD, is a noncompetitive antagonist of NMDA receptors that reduces Aβ toxicity by blocking abnormal activation of NMDA receptors, especially extrasynaptic NMDARs (Zadori et al., 2014).

In neurons, extrasynaptic NMDARs contain more NR2B subunits than NR2A subunits, and many cell death‐signaling proteins directly or indirectly bind to NR2B subunits (Zhou, Chen, Yun & Wang, 2015). PSD95, an adaptor of nNOS, can also link to extrasynaptic NMDARs. NR2B‐rich NMDARs suppress prosurvival CREB‐mediated gene expression by activating PSD95/nNOS signaling (Martel et al., 2012; Soriano et al., 2008). However, PSD95‐nNOS interaction is critical for synaptic connections (Nikonenko et al., 2008), and therefore, prolonged suppression of PSD95‐nNOS interaction may lead to unknown risks.

The carboxy‐terminal PDZ ligand of nNOS (NOS1AP, also named CAPON), a scaffolding protein of nNOS that indirectly binds to NMDARs, is a risk factor in many neurological diseases, such as schizophrenia, autism, bipolar disorder, post‐traumatic stress disorder, and depression (Candemir et al., 2016; Courtney, Li & Lai, 2014). CAPON forms a complex with nNOS and transmits NO signals to other proteins related to excitotoxicity when NMDARs are activated (Li et al., 2013; Zhu et al., 2015). CAPON also regulates dendritic morphology, dendrite patterning, and dendritic spine development (Candemir et al., 2016; Carrel et al., 2009; Richier et al., 2010). Both excitotoxicity and synaptic dysfunction are major causes of Alzheimer's disease. Thus, we speculated that nNOS–CAPON interaction might be an important downstream signaling pathway of abnormal NMDAR activation in Alzheimer's disease.

We previously reported that increased nNOS–CAPON interaction induced anxiety‐related behaviors *via* decreasing dendritic spine density and weakening prosurvival signals (Zhu et al., 2014). In this study, we found that nNOS–CAPON interaction was increased in both amyloid‐β_1‐42_‐treated primary cultured neurons *in vitro* and in the hippocampus of APP/PS1 mice. Blocking nNOS–CAPON interaction rescued neuron damage, the decrease in dendritic spines, memory loss, and prosurvival signals impaired by amyloid‐β. Therefore, our work presents a potential downstream signal of amyloid β‐mediated dysfunction of NMDARs.

## 2 RESULTS

### 2.1 nNOS–CAPON interaction was increased in primary cultured hippocampal neurons treated with Aβ and in the hippocampus of APP/PS1 mice

**Figure 1:**
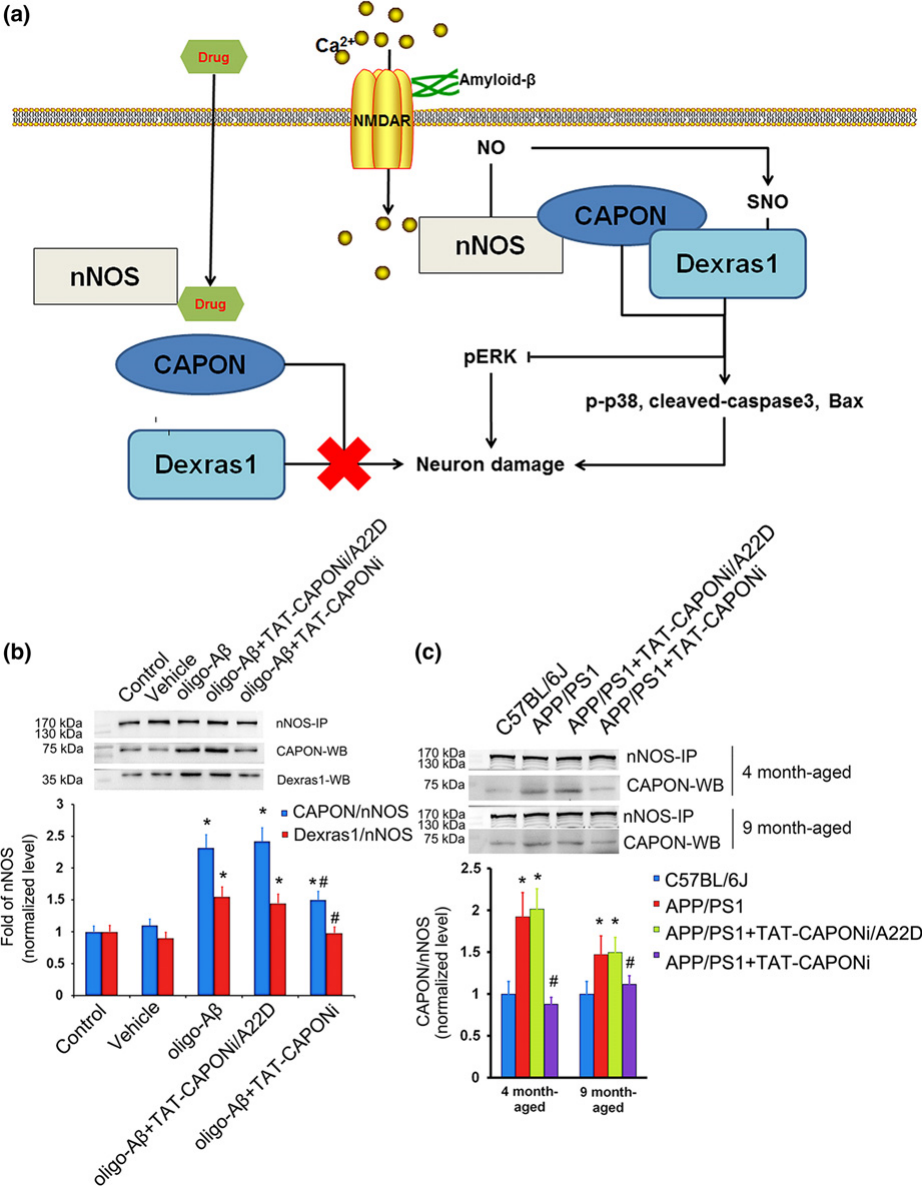
nNOS–CAPON interaction increased after exposure to oligo‐Aβ both *in vitro* and *in vivo*. (a) Summary of the role of nNOS–CAPON interaction in Aβ‐induced neurotoxicity. (b) Administration of oligo‐Aβ_1‐42_ influenced the nNOS–CAPON–Dexras1 interaction *in vitro* (*n *= 6, * *p *<* *.05 compared to the control group, # *p *<* *.05 compared to the oligo‐Aβ_1‐42_ group). (c) Interaction of nNOS–CAPON–Dexras1 increased in both 4‐month‐old and 9‐month‐old APP/PS1 mice compared to age‐matched wild‐type mice (*n *= 5, * *p *<* *.05 compared to the control group, # *p *<* *.05 compared to the APP/PS1 group)

As a potential ligand for NMDARs, Aβ may activate NMDARs and, consequently, increase nNOS–CAPON interaction, causing pathological changes in neurons (Candemir et al., 2016; Courtney et al., 2014). Here, we hypothesized that Aβ‐induced nNOS‐CPAON interaction was associated with Aβ neurotoxicity. Therefore, we incubated primary hippocampal neurons (DIV12) with 10 μmo/L oligo‐Aβ_1‐42_. Twenty‐four hours later, nNOS–CAPON interaction was evaluated by immunoprecipitation. The results showed that nNOS–CAPON interaction was increased significantly in the oligo‐Aβ_1‐42_ group compared with the vehicle group (Figure 1b, *p *< .05). APP/PS1 mice represent one of the most important animal models of Alzheimer's disease, and APP/PS1 mice show memory loss with age. nNOS–CAPON interaction was detected in the hippocampus of 4‐month‐old and 9‐month‐old APP/PS1 mice. Compared with aged‐matched background mice, APP/PS1 mice exhibited increased nNOS–CAPON interaction in the hippocampus (Figure 1c, *p *< .05). Therefore, nNOS–CAPON interaction was increased in the Alzheimer's disease model *in vitro* and *in vivo*. Subsequently, we determined whether increased nNOS–CAPON interaction contributed to Aβ‐mediated neurotoxicity. We constructed a peptide, named TAT‐CAPONi, to interfere with the nNOS–CAPON interaction. TAT‐CAPONi comprises a cell‐penetrating peptide (TAT) and the 12 amino acids of the C terminal of CAPON. TAT‐CAPONi selectively blocks nNOS–CAPON binding (Zhu et al., 2014). The Ala 22 Asp mutation of TAT‐CAPONi renders it incapable of binding to nNOS (Jaffrey, Snowman, Eliasson, Cohen & Snyder, 1998). Thus, we designed a mutated peptide, TAT‐CAPONi/A22D as a nonbinding control for TAT‐CAPONi. In the *in vitro* model of Alzheimer's disease and in APP/PS1 mice, the administration of TAT‐CAPONi reduced nNOS–CAPON interaction (Figure 1b, *p *< .05). Pretreatment with TAT‐CAPONi 45 min before the administration of oligo‐Aβ_1‐42_ also reduced neuronal damage *in vitro* (Fig. S1a, *p *< .05). Fibril‐Aβ_1‐42_, another toxicity form of Aβ_1‐42_, could also induce increased nNOS–CAPON interaction *in vitro* (Fig. S1c), and TAT‐CAPONi showed protective effects on neurotoxicity induced by fibril‐Aβ_1‐42_ (10 μmo/L) *in vitro* (Fig.  S1d). Together, these findings suggest that Aβ_1‐42_ causes an increase in nNOS–CAPON interaction, and nNOS–CAPON interaction contributes to neuronal damage induced by Aβ_1‐42_.

### 2.2 Blocking nNOS–CAPON interaction rescues memory deficits *in vivo*, especially in the early stage

**Figure 2:**
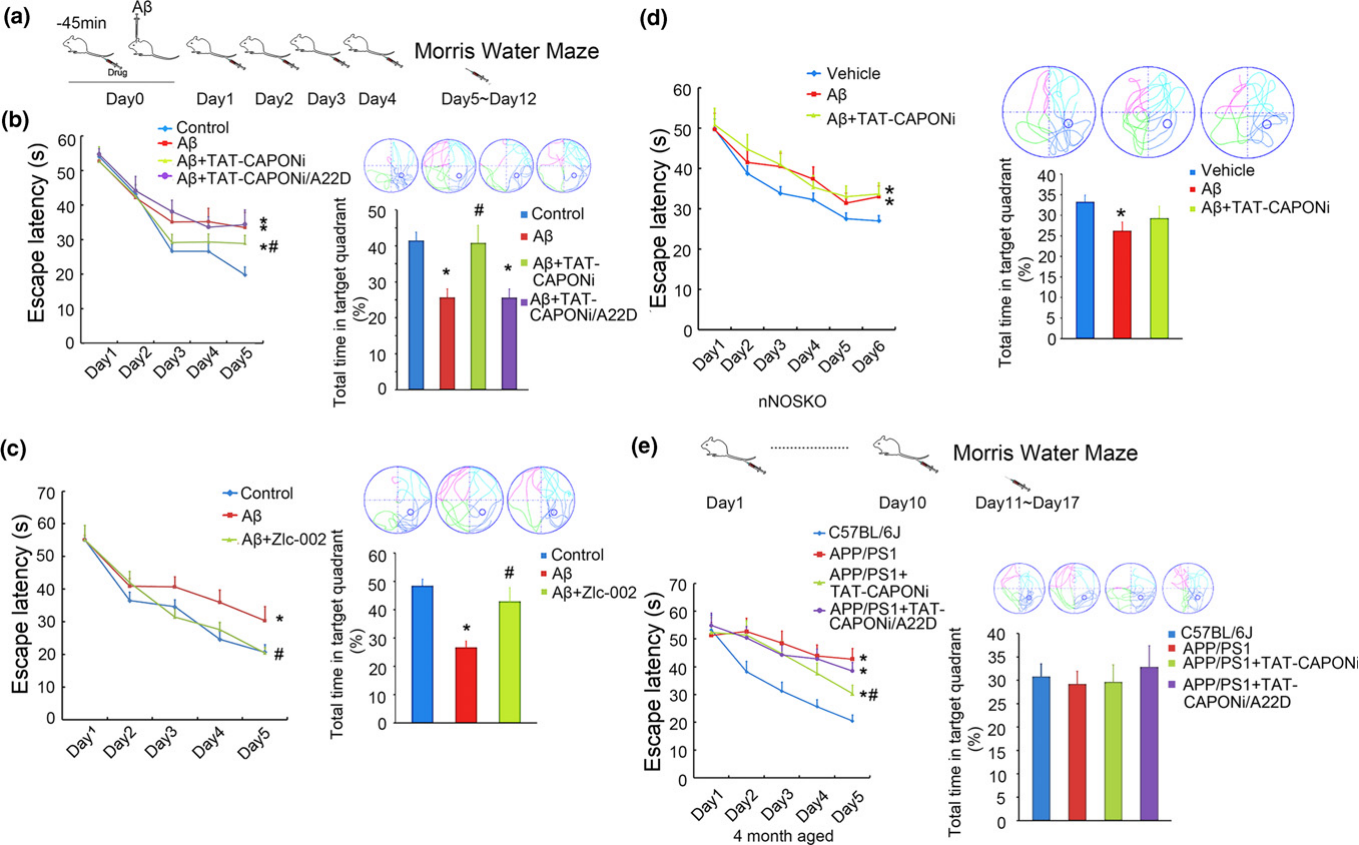
Blocking nNOS–CAPON interaction rescued memory loss *in vivo*. (a) Schedule implemented for the intracerebroventricular injection model. (b) After the injection of fibril‐Aβ_1‐42_, mice showed memory loss in the Aβ group, while administration with TAT‐CAPONi rescued memory loss. However, mice did not benefit from the administration of TAT‐CAPONi/A22D. (*n *= 14 per group in Morris water maze, * *p *<* *.05 compared to the control group, # *p *<* *.05 compared to the fibril‐Aβ_1‐42_ group). (c) Administration of Zlc‐002, a small molecule that blocks nNOS–CAPON interaction, rescued memory loss induced by Aβ_1‐42_ (*n *= 13 per group, * *p *<* *.05 compared to the control group, # *p *<* *.05 compared to the fibril‐Aβ_1‐42_ group)_._ (d) Blocking nNOS–CAPON interaction with TAT‐CAPONi could not rescue memory loss induced by Aβ_1‐42_ in nNOSKO mice (*n *= 14 per group). (e) Blocking nNOS–CAPON interaction rescued memory loss in 4‐month‐old APP/PS1 mice (*n *= 12 per group, * *p *<* *.05 compared to background mice, # *p *<* *.05 compared to the APP /PS1 group)

Injection of fibril‐Aβ_1‐42_ into the lateral ventricles of mice can induce Alzheimer's‐like symptoms (Shen, Yan & He, 2016). We pretreated C57BL/6J mice with TAT‐CAPONi or TAT‐CAPONi/A22D (3 nmol/g weight, i.v.) and injected fibril‐Aβ_1‐42_ into the lateral ventricles of mice after 45 min. Mice were treated with peptides at a dose of 3 nmol/g weight/day for 3 days, and their memory abilities were tested using the Morris water maze. TAT‐CAPONi, TAT‐CAPONi/A22D, or vehicle was administered to mice daily during the period of the Morris water maze test (Figure 2a). Injection of fibril‐Aβ_1‐42_ into the lateral ventricles of mice induced memory deficit (Figure 2b). There was no difference in the swimming speed of mice between groups (Fig. S2b). Administration of TAT‐CAPONi but not TAT‐CAPONi/A22D prevented the memory deficit (Figure 2b). To confirm the protective effects of uncoupling the nNOS–CAPON interaction, we repeated the above experiment with a small‐molecule blocker of nNOS–CAPON binding, Zlc002 (30 mg kg^−1^ day^−1^, i.v.) (Zhu et al., 2014). The results showed that Zlc002 rescued the memory loss of mice injected with Aβ (Figure 2c).

To exclude other potential pathways influenced by TAT‐CAPONi, we administered TAT‐CAPONi to nNOSKO mice and then injected fibril‐Aβ_1‐42_ into the lateral ventricles of the nNOSKO mice using the same protocol as above. nNOSKO mice did not benefit from the administration of TAT‐CAPONi in the Morris water maze test (Figure 2d), suggesting that the beneficial effects of TAT‐CAPONi are derived from the uncoupling nNOS and CAPON.

Finally, we evaluated the beneficial effect of TAT‐CAONi in APP/PS1 mice, a mice model widely used in Alzheimer's disease studies. APP/PS1 mice were treated with TAT‐CAPONi or TAT‐CAPONi/A22D via the tail vein for 5 days (3 nmol/g weight/day), and the memory of the mice was evaluated by the Morris water maze test. Mice were administered TAT‐CAPONi or TAT‐CAPONi/A22D (3 nmol/g weight) 45 min before the Morris water maze test each day. Administration of TAT‐CAPONi promoted memory function of 4‐month‐old APP/PS1 mice (Figure 2e), although there was no significant beneficial effect in 9‐month‐old APP/PS1 mice (Fig. S2c). Moreover, TAT‐CAPONi was able to across the blood brain barrier (Fig. S3).

In summary, blocking nNOS–CAPON interaction rescued the memory loss in the *in vivo* Alzheimer's disease model.

### 2.3 Blocking nNOS–CAPON interaction rescues dendritic impairments induced by Aβ

**Figure 3:**
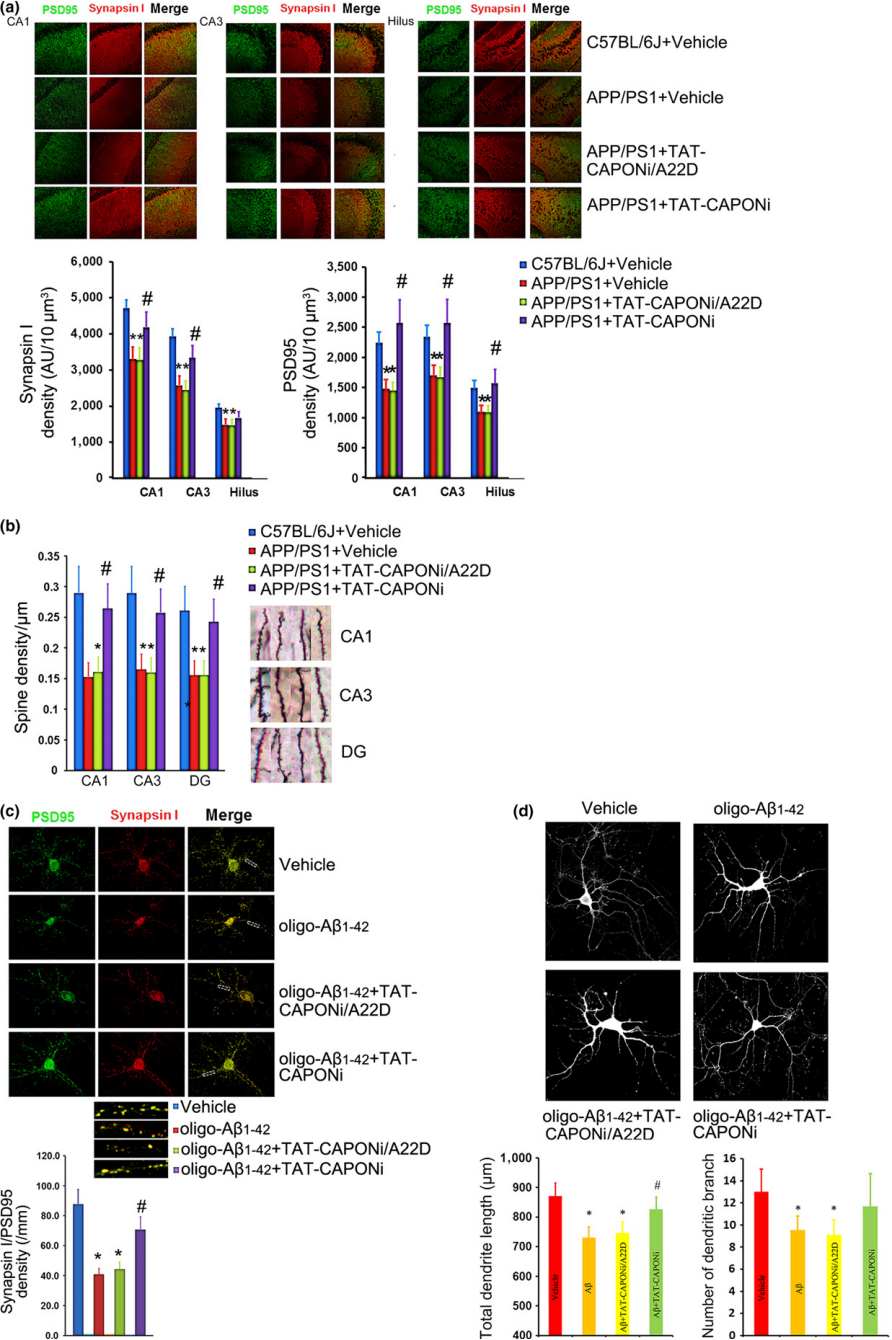
Blocking nNOS–CAPON interaction rescued dendritic changes both *in vivo* and *in vitro*. (a) Administration of TAT‐CAPONi increased the density of Synapsin I and PSD95 in the CA1, the CA3 and the hilus (5 mice per group, * *p *<* *.05 compared to the age‐matched C57BL/6J group, # *p *<* *.05 compared to the APP/PS1 group). (b) Administration of TAT‐CAPONi increased the density of spines in different regions of the hippocampus in APP/PS1 mice (*n *= 6, * *p *<* *.05 compared to the age‐matched C57BL/6J group, # *p *<* *.05 compared to the APP/PS1 group). (c,d) Administration of oligo‐Aβ_1‐42_ decreased the density of Synapsin I and PSD‐95 double‐labeling puncta in neurons, which indicated a loss of connections between neurons, but increased density of Synapsin I and PSD‐95 double‐labeling puncta was found after the administration of TAT‐CAPONi (*n *= 24 per group, * *p *<* *.05 compared to the control group, # *p* < .05 compared to the oligo‐Aβ_1‐42_ group). (e,f) Administration of TAT‐CAPONi rescued the length of dendrites *in vitro* (*n *= 30, * *p *<* *.05 compared to the control group, # *p *<* *.05 compared to the oligo‐Aβ_1‐42_ group)

Synaptic dysfunction and spine loss, but not neuron loss, are related to memory deficits in patients in the early stage of Alzheimer's disease and in young APP/PS1 mice (Herms & Dorostkar, 2016). Therefore, the beneficial effects observed upon blocking nNOS–CAPON interaction in the 4‐month‐old APP/PS1 mice might be due to rescue of synaptic function. To confirm this hypothesis, we evaluated morphological changes associated with dendritic and synaptic functions. First, we evaluated the PSD95 and Synapsin I densities in the hippocampus of APP/PS1 mice by immunofluorescence. PSD95 is a specific postsynaptic membrane protein, and Synapsin 1 is a specific presynaptic membrane protein. Therefore, the densities of PSD95 and Synapsin I partly reflect the potency of synaptic connections (Goetzl et al., 2016). The results showed that the densities of PSD95 and Synapsin I in the hippocampus of 4‐month‐old APP/PS1 mice were significantly lower than those of the background group, while the administration of TAT‐CAPONi increased the densities of both PSD95 and Synapsin I in the hippocampus of APP/PS1 mice (Figure 3a). Second, we evaluated the density of dendritic spines in the hippocampus by Golgi staining. The density of spines in the hippocampus was reduced in 4‐month‐old APP/PS1 mice compared with that in background mice, and TAT‐CAPONi increased the spine density (Figure 3b). Finally, we used a primary neuron model to confirm whether the change in dendrites was directly related to Aβ. Twenty‐four hours after neurons were treated with oligo‐Aβ_1‐42_, the densities of PSD95 and Synapsin 1 were significantly decreased, and the lengths and number of branch points of dendrites were also decreased (Figure 3c,d). Therefore, blocking nNOS–CAPON interaction with TAT‐CAPONi rescues dendritic impairments induced by Aβ *in vitro*.

### 2.4 ERK–CREB–BDNF pathway is involved in the effects of blocking nNOS–CAPON interaction

**Figure 4:**
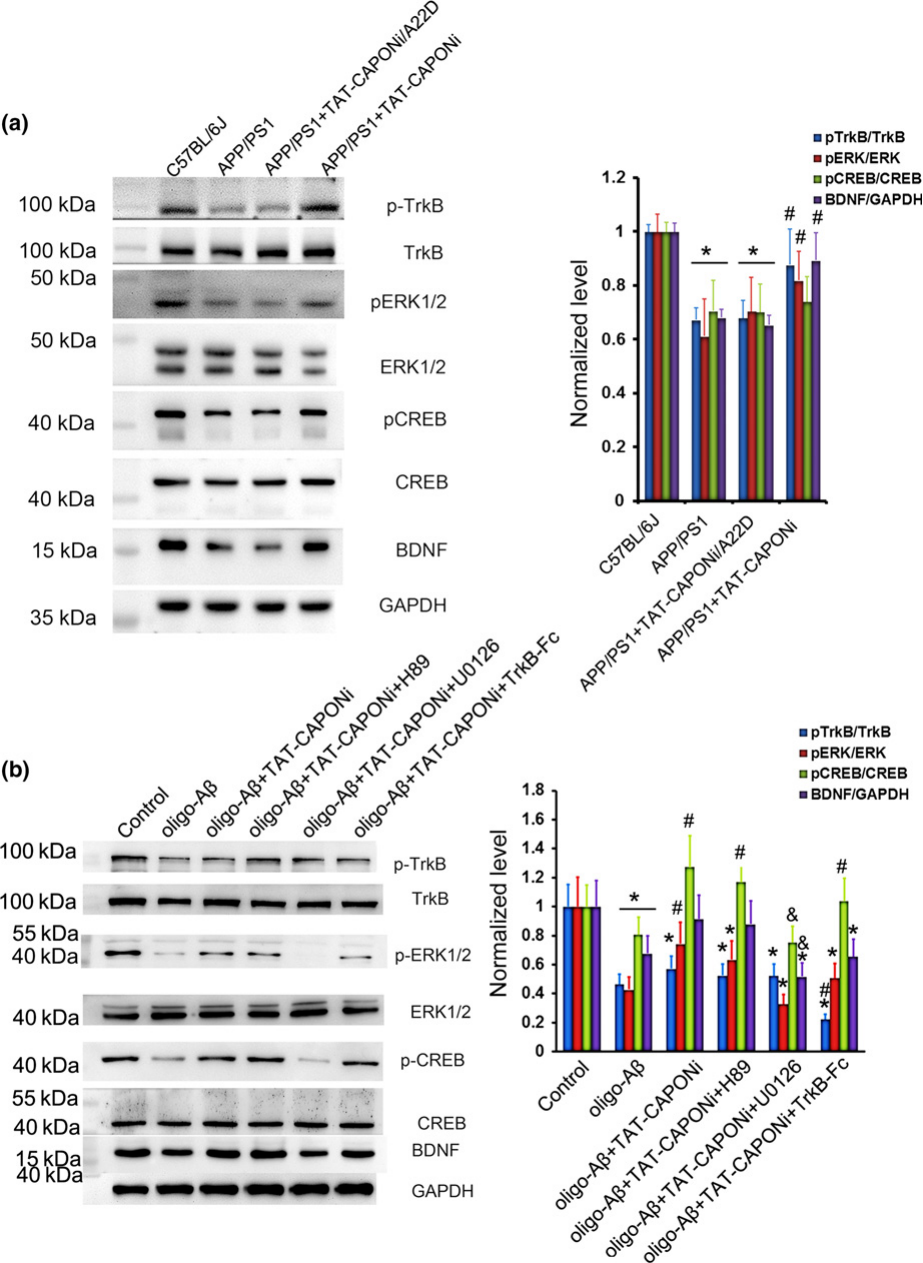
The beneficial effect of blocking the nNOS–CAPON interaction might be due to rescue of the ERK–CREB–BDNF pathway. (a) pression of spinophilin and BDNF and decreased phosphorylation of ERK and CREB in the hippocampus, and these changes were recovered by TAT‐CAPONi (*n *= 6, * *p *<* *.05 compared to the 4‐month‐old C57BL/6J mouse group, # *p *<* *.05 compared to the 4‐month‐old APP/PS1 mice group). (b) Beneficial effects could be observed *in vitro* and were abolished with an ERK1/2 inhibitor but not with a PKA inhibitor or a TrkB inhibitor (*n *= 6, * *p *<* *.05 compared to the control group, # *p *<* *.05 compared to the oligo‐Aβ group, & *p *<* *.05 compared to the oligo‐Aβ+TAT‐CAPONi group)

To explore the molecular mechanisms underlying the dendritic morphology changes after nNOS–CAPON blocking, we examined ERK phosphorylation, a kinase that influences synaptic maturation and stability (Collingridge, Peineau, Howland & Wang, 2010). TAT‐CAPONi significantly increased pERK abundance in the hippocampus of 4‐month‐old APP/PS1 mice (Figure 4a) and primary hippocampal neurons treated with oligo‐Aβ_1‐42_ (Figure 4b). Phosphorylation of CREB and expression of BDNF, two important signals relating to ERK, were also increased by blocking nNOS–CAPON interactions both *in vivo* and *in vitro* (Figure 4a,b). Subsequently, we determined the relationship of pERK, pCREB, and BDNF. U0126, a specific inhibitor of ERK phosphorylation, completely abolished almost all enhancements of the detected signals by TAT‐CAPONi treatment, including phosphorylation of CREB and TrkB and expression of BDNF (Figure 4b). The results suggested that ERK phosphorylation was an upstream signal necessary in the pathway. In addition to ERK, CREB can be phosphorylated by PKA. H89 is a specific inhibitor of PKA activity. Our data showed that H89 had no effects on the signal changes induced by TAT‐CAPONi (Figure 4b), excluding the role of PKA. To further determine the role of BDNF, we employed TrkB‐Fc, a specific inhibitor of the effect of BDNF. We found that ERK and CREB phosphorylation decreased in the oligo‐Aβ + TAT‐CAPONi + TrkB‐Fc group compared with in the oligo‐Aβ + TAT‐CAPONi group. However, CREB phosphorylation was still significantly higher in these groups than in the oligo‐Aβ group (Figure 4b). Therefore, blocking nNOS–CAPON interactions might decrease the inhibition of pERK by Aβ and then rescue the activity of CREB. Rescued phosphorylation of CREB increased the production of BDNF.

In conclusion, blocking nNOS–CAPON interaction can rescue dendritic changes and inhibition of the ERK–CREB–BDNF pathway induced by Aβ.

### 2.5 nNOS–CAPON‐Dexras1 complex and S‐nitrosylation of Dexras1 are related to neurotoxicity induced by Aβ

**Figure 5:**
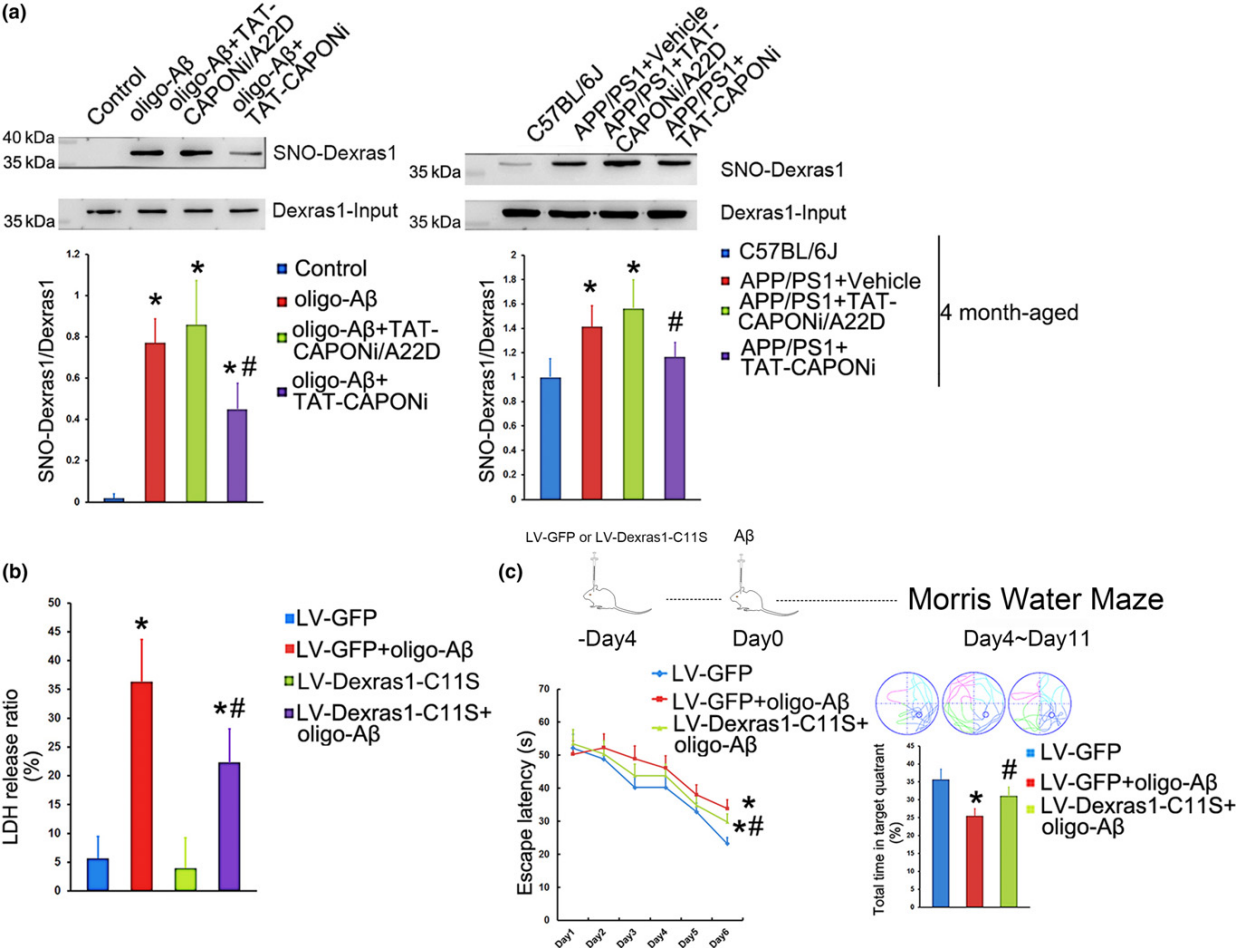
The nNOS–CAPON interaction mediated S‐nitrosylation of Dexras1, which plays important roles in the neurotoxicity induced by Aβ. (a) Increased S‐nitrosylation of Dexras1 was detected *in vitro* and *in vivo*, but the increase could be rescued by administration of TAT‐CAPONi (*n *= 6 *in vitro*,* n *= 4 in the model *in vivo*, * *p *<* *.05 compared to the control group, # *p *<* *.05 compared to the oligo‐Aβ_1‐42_ group or the APP/PS1 group). (b) Overexpression of Dexras1‐C11S, in which an S‐nitrosylation site is lost, decreased the neuronal injury induced by Aβ_1‐42_ (*n *= 6, * *p *<* *.05 compared to the LV‐GFP group, # *p *<* *.05 compared to the LV‐GFP+ oligo‐Aβ_1‐42_ group). (c) Overexpression of Dexras1‐C11S also rescued memory loss in the *in vivo* intracerebroventricular injection model (*n *= 12 per group, * *p *<* *.05 compared to the control group, # *p *<* *.05 compared to the fibril‐Aβ_1‐42_ group)

In our previous study, we reported that nNOS–CAPON interaction contributed to anxiety via Dexras1 (Zhu et al., 2014). The formation of nNOS–CAPON–Dexras1 and the S‐nitrosation of Dexras1 also mediate the neurotoxicity of NMDARs (Cheah et al., 2006). Dexras1 was increased in the ternary complex of nNOS–CAPON–Dexras1 after the stimulation of oligo‐Aβ_1‐42_ (Figure 1b). Thus, we questioned whether Dexras1 can be activated *via* nNOS–CAPON–Dexras1 signaling during Aβ treatment. We indeed detected increased S‐nitrosylation of Dexras1 in both primary hippocampal neurons after oligo‐Aβ_1‐42_ stimulation and in the hippocampus of 4‐month‐old APP/PS1 mice (Figure 5a). In addition, blocking nNOS–CAPON interaction not only decreased the formation of nNOS–CAPON–Dexras1 but also the S‐nitrosylation of Dexras1 (Figure 5a). Moreover, these changes were partially reversed by blocking nNOS–CAPON interaction (Figure 5a), which was consistent with decreased levels of nNOS–CAPON–Dexras1 complex (Figure 1b). Thus, the S‐nitrosylation of Dexras1 was involved in Aβ neurotoxicity. To further test this hypothesis, we generated a lentivirus overexpressing Dexras1, containing a Cys 11 Ser mutation (named LV‐Dexras1‐C11S). This mutant Dexras1 cannot be activated by S‐nitrosylation (Cheah et al., 2006). Neurons overexpressing Dexras1‐C11S showed tolerance to neurotoxicity induced by oligo‐Aβ_1‐42_ *in vitro* (Figure 5b).

To investigate whether S‐nitrosylation of Dexras1 mediated memory impairment induced by Aβ, we injected LV‐Dexras1‐C11S or LV‐GFP into the hippocampus of C57BL/6J mice. Four days later, fiber‐Aβ_1‐42_ was injected into the lateral ventricle of C57BL/6J mice, and after another four days, the mice were subjected to the Morris water maze test. Aβ impaired the learning and memory abilities of the mice, but these impairments were significantly ameliorated in the group expressing Dexras1‐C11S comparing with the empty virus group (Figure 5c).

*In vitro* and *in vivo* results showed that formation of the nNOS–CAPON–Dexras1 complex and S‐nitrosylation of Dexras1 could be induced by Aβ, and either blocking the formation of nNOS–CAPON–Dexras1 or the S‐nitrosylation of Dexras1 protected neurons from the Aβ neurotoxicity. Thus, Dexras1 should be a part of the toxicity signal mediated by nNOS–CAPON interaction.

Based on these results, we demonstrated that neurotoxicity mediated by Aβ is associated with nNOS–CAPON interaction. Furthermore, the results indicate that the ERK–CREB–BDNF signaling pathway and S‐nitrosation of Dexras1 are likely downstream of nNOS–CAPON interaction.

## 3 DISCUSSION

The amyloid cascade hypothesis is one of the most prominent theories in Alzheimer's disease research, while Aβ_1‐42_ and/or its oligomers are thought as the major toxic components. Clinical evidence suggests that increases in Aβ_1‐42_ and its oligomers are associated with disease, and laboratory evidence suggests that Aβ_1‐42_ oligomers induce abnormal synaptic function and neuronal death (Wilcox, Lacor, Pitt & Klein, 2011). Based on the amyloid cascade hypothesis, studies of Alzheimer’ disease have generally focused on two common treatment strategies: 1. decreasing the level of Aβ, especially toxic forms; and 2. protecting neurons or maintaining neuronal function.

Inhibition/activation of secretases of Aβ generation in the amyloidogenic pathway was shown to significantly decrease the level of Aβ (Parsons & Rammes, 2017), but some forms of Aβ were reported to protect the brain from infection and inflammation (Kumar et al., 2016). In physiological conditions, some transmembrane receptors and signaling proteins are also substrates of alpha‐, beta‐, and gamma‐secretase, key enzymes in the amyloidogenic pathway, and thus interference with the activation of secretases causes serious side effects (Parsons & Rammes, 2017). Therefore, strategies aimed to decrease the total level of Aβ may be debatable. Neutralizing toxic forms of Aβ with antibodies seems to be a more direct approach than interfering with secretases, but the outcomes of clinical trials using antibody drugs showed less beneficial effects than expected (Freskgard & Urich, 2017). Drugs are also developed to modulate Aβ aggregation, but a more detailed understanding of the molecules that retard aggregate formation and of the structure‐activity relationships are needed (Alam, Siddiqi, Chturvedi & Khan, 2017). Therefore, many problems must be solved before drugs can be used to treat Alzheimer's disease by decreasing Aβ or its toxic forms.

Another strategy is to reduce the toxicity of Aβ based on its target receptors or proteins. Although a number of receptors and target proteins have been studied in Alzheimer's disease (Ul Islam, Khan, Jabir, Kamal & Tabrez, 2017), the only drugs approved for clinical treatment are still acetylcholinesterase inhibitors and memantine. Clinical and preclinical studies of memantine have proven that it confers beneficial effects by inhibiting NMDARs, and NMDARs are relatively mature targets in Alzheimer's disease. However, most NMDAR inhibitors have failed in clinical trials because they also inhibit the normal function of NMDARs in neurons. Therefore, selectively blocking excitotoxicity mediated by NMDARs has become an important goal in designing NMDAR inhibitors. In addition to NMDARs themselves, downstream molecules of NMDARs are also targets of drug design, and inhibiting nNOS showed protective effects in Alzheimer's disease model mice (Misra, Kuhad & Chopra, 2013; Yu et al., 2013). However, nNOS and the NO produced by nNOS play important roles in the normal function of neurons (Zhou & Zhu, 2009). Nonetheless, no nNOS inhibitor has yet to be approved for entry into clinical trials.

In the present research, we focused on nNOS–CAPON interaction, which is a downstream signal of nNOS and NMDARs. According to previous studies, increased nNOS–CAPON interaction mediates excitotoxicity, overload of iron, and stress‐related depressive behaviors in the brain (Courtney et al., 2014). Under physiological conditions, CAPON regulates dendrite patterning and dendritic spine development of neurons, and Dexras1 plays important roles in the circadian clock and the responses to fluid deprivation and salt loading (Carrel et al., 2009; Cheng et al., 2004; Greenwood et al., 2016; Richier et al., 2010; Van Gelder, 2004). These functions are associated with neither nNOS–CAPON interaction nor nNOS. Moreover, nNOS–CAPON interaction blockers used in our experiments did not affect the resting membrane potential of neurons, the interaction of PSD95 with NMDARs, or the interaction of CAPON with synapsins (Zhu et al., 2014). Therefore, blocking nNOS–CAPON interaction might induce fewer side effects than the direct intervention of NMDARs or nNOS. The ERK–CREB–BDNF pathway is important for maintaining the survival and function of neurons in Alzheimer's disease, and increased activation of this pathway might slow the development of Alzheimer's disease (Kamat et al., 2016). The beneficial effects of blocking nNOS–CAPON interaction come from the recovery of dendrites and the ERK–CREB–BDNF pathway, and thus, this approach is different from blocking NMDARs, such as with memantine, which decreases the influx of Ca^2+^ and excitotoxicity (Ferreira‐Vieira, Guimaraes, Silva & Ribeiro, 2016). The difference of plaque deposition between 4‐month‐old APP/PS1 mice than in 9‐month‐old APP/PS1 mice (Fig. S4a) might be the reason that blocking nNOS–CAPON interaction showed beneficial effects in 4‐month‐old APP/PS1 mice but not in 9‐month‐old APP/PS1 mice. nNOS–CAPON blockers combined with memantine, a NMDAR blocker used in severe Alzheimer's disease, might confer more beneficial effects than memantine or nNOS–CAPON blockers alone, especially in the early stage of disease.

Conceivably, increased nNOS–CAPON interaction mediated the dysfunction of neurons in the Alzheimer's disease model mice, and the nNOS–CAPON interaction may be a new target for developing drugs to treat Alzheimer's disease.

## 4 EXPERIMENTAL PROCEDURES

### 4.1 Ethical statements of animal research

Homozygous male nNOS‐deficient mice (B6; 129S4*‐Nos1* ^*tm1Plh*^, nNOS^−/−^; The Jackson Laboratory) and male wild‐type (WT) mice with a similar genetic background (B6129SF1, nNOS^+/+^) were maintained in the Model Animal Research Center of Nanjing University. The nNOS^−/−^ and nNOS^+/+^ embryos were used for cell cultures. Male C57BL/6J‐TgN (APP/PS1) and male WT mice (C57BL/6J) were purchased from Beijing HFK Bio‐technology Co. Ltd. APP/PS1 mice express chimeric amyloid precursor protein (APPswe) encoding the Swedish mutations K595N/M596L, and human presenilin 1 PS1‐dE9 (deletion of exon 9) controlled by independent mouse prion protein promoter elements. All experimental protocols using animals were approved by the Institutional Animal Care and Use Committee of Nanjing Medical University.

### 4.2 Reagents

Rabbit anti‐NOS1AP (sc‐9138) and mouse antigreen fluorescent protein (sc‐101525) were purchased from Santa Cruz (USA, Dallas). Rabbit anti‐nNOS (#4231), β‐amyloid (1‐42 Specific) (D9A3A) rabbit mAb #14974, Synapsin‐1 (D12G5) XP^®^ Rabbit mAb (#5297), rabbit anti‐CREB (#9197), rabbit anti‐ERK1/2 (#4695), mouse anti‐pERK1/2 (#5726), and mouse anti‐pCREB (#9196) were purchased from CST (Danvers, MA). Rabbit anti‐BDNF (ab108319), mouse anti‐HIV1 tat antibody [N3] (ab63957), rabbit anti‐TrkB (ab187041), and rabbit anti‐pTrkB (ab109684) were purchased from Abcam (Cambridge MA). Rabbit anti‐Dexras1 (ab15794) and mouse anti‐PSD95 (MAB1596) were purchased from Millipore (Danvers, MA). Zlc002, a small‐molecule inhibitor of nNOS–CAPON interaction, was designed and synthesized in our laboratory (Zhu et al., 2014). H89 (HY‐15979) and U0126 (HY‐12031) were purchased from MedChem Express. TrkB‐Fc was purchased from Sino Biological. Other chemical reagents were purchased from Sigma‐Aldrich and Thermo Fisher. TAT‐CAPONi (YGRKKRRQRRRELGDSLDDEIAV), TAT‐CAPONi/A22D (YGRKKRRQRRRELGDSLDDEIDV), and amyloid‐β_1‐42_ (purity >95%) were purchased from GL Biochem (Shanghai) Ltd.

### 4.3 Cell cultures

Primary neurons were isolated from the hippocampus of mouse embryos at embryonic day 15 (E15), as previously described (Zhang et al., 2010). Cultures were maintained in neurobasal media supplemented with B27, penicillin, streptomycin, and L‐glutamine. Cells were cultured on polyornithine‐coated tissue culture dishes (3.5 cm in diameter) at a density of 1 × 10^4^ cells/cm^2^ (morphological analysis) or 1 × 10^5^ cells/cm^2^ (morphological analysis). Cells were grown for 7–14 d *in vitro* (DIV) and used for specific experiments, as indicated below. The proportion of β‐III‐tubulin^+^ cells at 10 DIV was ∼92%.

All cultures were maintained in an incubator (HERAcell 150, Thermo Fisher Scientific) with a humidified atmosphere of 95% air and 5% CO_2_ at 37°C.

### 4.4 Preparation of Aβ_1–42_ oligomers and fibrils

Oligomerized Aβ_1–42_ was prepared as previously reported (Brkic et al., 2015). Briefly, Aβ_1–42_ was dissolved at 1 mg/ml in hexafluoroisopropanol (HFIP; Sigma‐Aldrich), and the HFIP was removed after 1 hr. The peptide film was resolved at 1 mg/ml in DMSO (Sigma‐Aldrich). The solution was diluted to 100 μmol/L with Ham's F‐12 medium without glutamine. Then, the solution was allowed to stand for 1 hr at 25°C followed by centrifuging at 14,000 × *g* for 10 min at 4°C to remove any insoluble aggregates.

For preparation of fibrils, the dried hexafluoro‐2‐propanol film of Aβ_1–42_ (see above) was dissolved in sterile PBS to yield a 3 μg/μl solution and incubated for 7 days at 37°C (De Felice et al., 2007).

### 4.5 Intrahippocampal and intracerebroventricular injections

One microliter of lentivirus (~10^6^ virus particles) was bilaterally injected into the hippocampus at a rate of 0.5 μl/min. The needle was left in place for 3 min after the injection and then slowly retracted, followed by cleaning and suturing of the wound. The injection was performed at the following coordinates: AP: −2.1 mm, ML: ±1.3 mm, DV: −2.3 mm. Three microliters of Aβ_1‐42_ (9 μg) was injected i.c.v. at a rate of 0.5 μl/min. The needle was left in place for 3 min after the injection and then slowly retracted, followed by cleaning and suturing of the wound. Sham mice received the equivalent volume of PBS into the ventricle. The injection was performed at the following coordinates: AP: −0.6 mm, ML: ±1.2 mm, DV: −2 mm.

### 4.6 Recombinant virus production and infection

The recombinant lentivirus LV‐GFP‐Dexras1‐C11S or its control LV‐GFP was generated as we previously described (Zhu et al., 2014). Cultured neurons were infected with LV‐GFP‐Dexras1‐C11S or LV‐GFP containing 1.0 × 10^9^ transduction units/ml at day 4 *in vitro* [multiplicity of infection (MOI) = 2.5]. The medium was half changed 8 hr later and fully changed 24 hr later.

### 4.7 Western blot analysis

Western blot analysis was performed as described previously. The primary antibodies were as follows: rabbit anti‐CAPON (1:500), rabbit anti‐ERK1/2 (1:500), rabbit anti‐CREB (1:500), rabbit anti‐nNOS (1:1000), mouse anti‐pERK1/2 (1:500), mouse anti‐pCREB (1:1000), rabbit anti‐BDNF (1:1000), rabbit anti‐Dexras1 (1:1000), and rabbit anticleaved‐caspase 3 (1:400). An internal control was performed using mouse anti‐GAPDH (1:2000; KangChen Bio‐tech) or mouse anti‐β‐actin (1:2000; KangChen Bio‐tech), and appropriate horseradish peroxidase‐linked secondary antibodies were used for enhanced chemiluminescence detection (Pierce).

### 4.8 Coimmunoprecipitation

Lysis and coimmunoprecipitation of cultures and tissues were performed as we previously described (Zhou et al., 2010). Cultured neurons or hippocampal tissues were lysed in 50 mmol/L Tris–HCl (pH 7.4) in buffer containing 150 mmol/L NaCl, 1 mmol/L EDTA‐Na, 1% NP‐40, 0.02% sodium azide, 0.1% SDS, 0.5% sodium deoxycholate, 1% PMSF, 1‰ aprotinin, 1‰ leupeptin, and 0.5‰ pepstatin A. The lysates were centrifuged at 12,000 × *g* for 15 min at 4°C. The supernatant (200 μl) was preincubated for 1 h at 4°C with 25 μl of protein G‐Sepharose beads (Sigma‐Aldrich) and then centrifuged to remove proteins that adhered nonspecifically to the beads and to obtain the target supernatant for the following IP experiment. Protein G‐Sepharose beads were incubated with rabbit anti‐nNOS (1:200) for 3–4 h. The antibody‐conjugated protein G‐Sepharose beads and the target supernatant were added for incubation overnight at 4°C. Immune complexes were isolated by centrifuging and washing five times with 0.05 mol/L HEPES buffer (pH 7.1) containing 0.15% Triton X‐100, 0.15 mol/L NaCl, and 0.1 × 10^−3 ^mol/L sodium orthovanadate. Bound proteins were eluted by heating at 100°C in loading buffer. Proteins were analyzed by immunoblotting using rabbit anti‐CAPON (1:500) or rabbit anti‐nNOS (1:1000).

### 4.9 Morris water maze

The spatial cognitive performance of mice was evaluated by the Morris water maze. The Morris water maze protocol has been described in detail in our previous report (Li et al., 2009). In brief, the mouse was placed in opaque water of a circular swimming pool and trained to locate the hidden platform 0.5 cm under the surface of the water. During the training to find the hidden platform, mice were allowed to swim for a maximum of 60 s in the pool for each trial. One block of four trials per day was performed for 5 consecutive days. On the sixth day, mice performed one 60 s retention probe test during which the platform was removed from the pool. During retention, the number of crossings of the platform location and the time spent in the target quadrant were measured.

### 4.10 Biotin‐switch assay

This assay was performed in the dark, as previously described (Fang et al., 2000). Briefly, cells were lysed in HEN buffer (250 mmol/L HEPES, 1 mmol/L EDTA, and 100 mmol/L neocuproine) adjusted to contain 0.4% CHAPS. Samples were homogenized, and free cysteines were blocked for 1 hr at 50°C in three volumes of blocking buffer [HEN buffer plus 2.5% SDS (HENS)] containing methyl methanethiosulfonate (200 mmol/L; Sigma‐Aldrich). Proteins were precipitated with acetone at −20°C and resuspended in 300 μl of HENS solution. After adding fresh ascorbic acid (20 mmol/L; Sigma‐Aldrich) and biotin‐HPDP [*N*‐[6‐(biotinamido)hexyl]‐3‐pyridyldithio)‐propionamide; 1 mmol/L; Sigma‐Aldrich], proteins were incubated at room temperature for 1 hr. After separation using an SDS–PAGE gel in nonreducing loading buffer, biotinylated proteins were detected by immunoblotting using rabbit antibiotin. Alternatively, biotinylated proteins were resuspended in 250 μl of HENS buffer plus 500 μl of neutralization buffer (20 mmol/L HEPES, 100 mmol/L NaCl, 1 mmol/L EDTA, 0.5% Triton X‐100) and precipitated with 50 μl of prewashed avidin‐affinity resin beads (Sigma‐Aldrich) at room temperature for 1 h. The beads were washed five times at 4°C using neutralization buffer containing 600 nmol/L NaCl. Biotinylated proteins were eluted using 30 μl of elution buffer (20 mmol/L HEPES, 100 mmol/L NaCl, 1 mmol/L EDTA, 100 mmol/L β‐mercaptoethanol) and heated at 100°C for 5 min in reducing SDS–PAGE loading buffer.

### 4.11 Golgi‐Cox staining

Fresh brains that had not undergone perfusion or fixation were used for Golgi‐Cox staining to show subtle morphological alterations in neuronal dendrites and dendritic spines. Golgi‐Cox staining was performed with an FD Rapid GolgiStain Kit (FD NeuroTechnologies) according to the user manual. Briefly, the brains were first placed in impregnation solution for 2 weeks followed by 2 days in a 30% sucrose solution. Then, they were cut into 100 μm coronal sections using a vibratome (World Precision Instruments) and stained. For morphological analysis, 10 random neurons from each sample were measured, and the average was regarded as the final value of one sample.

### 4.12 Immunofluorescence

The details of immunofluorescence for brain sections and cultured cells have been described (Luo et al., 2014). Briefly, brain slices were fixed in 4% paraformaldehyde (30 μm) and cultured cells were blocked with blocking solution (10% serum of donkey, 0.2% Triton X‐100). After washing with PBS, samples were incubated with primary antibodies overnight at 4°C. Then, samples were washed with PBS and incubated with secondary antibodies (2 hr at room temperature). After washing with PBS, samples were counterstained with Hoechst 33258 (Sigma‐Aldrich) to label the nuclei and mounted. Fluorescence was visualized by confocal microscopy (LSM 700, Zeiss). The primary antibodies used were as follows: mouse/rabbit anti‐GFP (1:500), rabbit anti‐Synapsin‐1 (1:500), and mouse anti‐PSD95 (1:500). Secondary antibodies used were goat anti‐mouse Dylight 488 (1:200; Jackson ImmunoResearch) and goat anti‐rabbit Cy3 (1:400; Jackson ImmunoResearch).

For the immunofluorescence analysis of brain sections from TAT‐CAPONi‐treated mice and control mice, brains were obtained 45 min after the injection (TAT‐CAPONi or vehicle) and fixed with 4% paraformaldehyde. After sectioning, brain slices (30 μm) were treated with goat anti‐mouse IgG (H+L) (1:200) at 30°C for 1 hr after blocking with blocking solution. After washing with PBS, samples were incubated with anti‐HIV1 tat antibody (Abcam, ab63957, 1:100, overnight at 4°C). Then, samples were washed with PBS and incubated with secondary antibodies (2 hr at room temperature). After washing with PBS, samples were counterstained with DAPI (Sigma‐Aldrich) to label the nuclei and mounted. Fluorescence was visualized by confocal microscopy.

### 4.13 Congo red staining

Brain slices (30 μm) fixed in 4% paraformaldehyde were used for Congo red staining. For Congo red staining, the sections were stained in Congo red (0.5% in methanol with 20% glycerol) for 20 min, differentiated in alkaline alcohol solution, counterstained with Gill's hematoxylin for 30 s, and finally mounted with neutral gum. Congo red‐stained plaques were visualized by microscopy.

### 4.14 Statistical analysis

Comparisons among multiple groups were made with one‐way ANOVA (one factor) or two‐way ANOVA (two factors) followed by Scheffé's *post hoc* test. Comparisons between two groups were made with two‐tailed Student's *t* test. Data were presented as the mean ± SEM, and *p *< .05 was considered statistically significant. Investigators were blinded to the group allocation when assessing the outcomes.

## AUTHOR CONTRIBUTIONS

Yu Zhang participated in designing the study, supervising the analysis, and writing the manuscript. Zhu Zhu, Hai‐Ying Liang, and Lei Zhang carried out the analysis and participated in designing the study. Huan‐Yu Ni participated in the analysis. Qi‐Gang Zhou participated the progress of the revision and helped to write the manuscript. Chun‐Xia Luo and Dong‐Ya Zhu participated in the study design, coordinating the study, and drafting and finalizing the manuscript.

## CONFLICT OF INTEREST

All authors read and approved the final manuscript and declare no conflict of interest.

## References

[acel12754-bib-0001] Alam, P. , Siddiqi, K. , Chturvedi, S. K. , & Khan, R. H. (2017). Protein aggregation: From background to inhibition strategies. International Journal of Biological Macromolecules, 103, 208–219. 28522393 10.1016/j.ijbiomac.2017.05.048

[acel12754-bib-0002] Brkic, M. , Balusu, S. , Van Wonterghem, E. , Gorle, N. , Benilova, I. , Kremer, A. , … Vandenbroucke, R. E. (2015). Amyloid beta oligomers disrupt blood‐CSF barrier integrity by activating matrix metalloproteinases. Journal of Neuroscience, 35, 12766–12778. 26377465 10.1523/JNEUROSCI.0006-15.2015 PMC6795210

[acel12754-bib-0003] Candemir, E. , Kollert, L. , Weissflog, L. , Geis, M. , Muller, A. , Post, A. M. , … Freudenberg, F. (2016). Interaction of NOS1AP with the NOS‐I PDZ domain: Implications for schizophrenia‐related alterations in dendritic morphology. European Neuropsychopharmacology, 26, 741–755. 26861996 10.1016/j.euroneuro.2016.01.008

[acel12754-bib-0004] Carrel, D. , Du, Y. , Komlos, D. , Hadzimichalis, N. M. , Kwon, M. , Wang, B. , … Firestein, B. L. (2009). NOS1AP regulates dendrite patterning of hippocampal neurons through a carboxypeptidase E‐mediated pathway. Journal of Neuroscience, 29, 8248–8258. 19553464 10.1523/JNEUROSCI.5287-08.2009 PMC2819070

[acel12754-bib-0005] Cheah, J. H. , Kim, S. F. , Hester, L. D. , Clancy, K. W. , Patterson, S. E. 3rd , Papadopoulos, V. , & Snyder, S. H. (2006). NMDA receptor‐nitric oxide transmission mediates neuronal iron homeostasis via the GTPase Dexras1. Neuron, 51, 431–440. 16908409 10.1016/j.neuron.2006.07.011 PMC3150500

[acel12754-bib-0006] Cheng, H. Y. , Obrietan, K. , Cain, S. W. , Lee, B. Y. , Agostino, P. V. , Joza, N. A. , … Penninger, J. M. (2004). Dexras1 potentiates photic and suppresses nonphotic responses of the circadian clock. Neuron, 43, 715–728. 15339652 10.1016/j.neuron.2004.08.021

[acel12754-bib-0007] Collingridge, G. L. , Peineau, S. , Howland, J. G. , & Wang, Y. T. (2010). Long‐term depression in the CNS. Nature Reviews Neuroscience, 11, 459–473. 20559335 10.1038/nrn2867

[acel12754-bib-0008] Courtney, M. J. , Li, L. L. , & Lai, Y. Y. (2014). Mechanisms of NOS1AP action on NMDA receptor‐nNOS signaling. Front Cell Neurosci, 8, 252. 25221472 10.3389/fncel.2014.00252 PMC4145862

[acel12754-bib-0009] De Felice, F. G. , Velasco, P. T. , Lambert, M. P. , Viola, K. , Fernandez, S. J. , Ferreira, S. T. , & Klein, W. L. (2007). Abeta oligomers induce neuronal oxidative stress through an N‐methyl‐D‐aspartate receptor‐dependent mechanism that is blocked by the Alzheimer drug memantine. Journal of Biological Chemistry, 282, 11590–11601. 17308309 10.1074/jbc.M607483200

[acel12754-bib-0010] Fang, M. , Jaffrey, S. R. , Sawa, A. , Ye, K. , Luo, X. , & Snyder, S. H. (2000). Dexras1: a G protein specifically coupled to neuronal nitric oxide synthase via CAPON. Neuron, 28, 183–193. 11086993 10.1016/s0896-6273(00)00095-7

[acel12754-bib-0011] Ferreira‐Vieira, T. H. , Guimaraes, I. M. , Silva, F. R. , & Ribeiro, F. M. (2016). Alzheimer's disease: targeting the cholinergic system. Current Neuropharmacology, 14, 101–115. 26813123 10.2174/1570159X13666150716165726 PMC4787279

[acel12754-bib-0012] Freskgard, P. O. , & Urich, E. (2017). Antibody therapies in CNS diseases. Neuropharmacology, 120, 38–55. 26972827 10.1016/j.neuropharm.2016.03.014

[acel12754-bib-0013] Goetzl, E. J. , Kapogiannis, D. , Schwartz, J. B. , Lobach, I. V. , Goetzl, L. , Abner, E. L. , … Miller, B. L. (2016). Decreased synaptic proteins in neuronal exosomes of frontotemporal dementia and Alzheimer's disease. FASEB J, 30, 4141–4148. 27601437 10.1096/fj.201600816R PMC5102122

[acel12754-bib-0014] Greenwood, M. P. , Greenwood, M. , Mecawi, A. S. , Antunes‐Rodrigues, J. , Paton, J. F. , & Murphy, D. (2016). Rasd1, a small G protein with a big role in the hypothalamic response to neuronal activation. Mol Brain, 9, 1. 26739966 10.1186/s13041-015-0182-2 PMC4704412

[acel12754-bib-0015] Herms, J. , & Dorostkar, M. M. (2016). Dendritic spine pathology in neurodegenerative diseases. Annual Review of Pathology: Mechanisms of Disease, 11, 221–250. 10.1146/annurev-pathol-012615-044216 26907528

[acel12754-bib-0016] Jaffrey, S. R. , Snowman, A. M. , Eliasson, M. J. , Cohen, N. A. , & Snyder, S. H. (1998). CAPON: a protein associated with neuronal nitric oxide synthase that regulates its interactions with PSD95. Neuron, 20, 115–124. 9459447 10.1016/s0896-6273(00)80439-0

[acel12754-bib-0017] Jarosz‐Griffiths, H. H. , Noble, E. , Rushworth, J. V. , & Hooper, N. M. (2016). Amyloid‐beta receptors: the good, the bad, and the prion protein. Journal of Biological Chemistry, 291, 3174–3183. 26719327 10.1074/jbc.R115.702704 PMC4751366

[acel12754-bib-0018] Kamat, P. K. , Kalani, A. , Rai, S. , Swarnkar, S. , Tota, S. , Nath, C. , & Tyagi, N. (2016). Mechanism of oxidative stress and synapse dysfunction in the pathogenesis of Alzheimer's Disease: understanding the therapeutics strategies. Molecular Neurobiology, 53, 648–661. 25511446 10.1007/s12035-014-9053-6 PMC4470891

[acel12754-bib-0019] Kumar, D. K. , Choi, S. H. , Washicosky, K. J. , Eimer, W. A. , Tucker, S. , Ghofrani, J. , … Moir, R. D. (2016). Amyloid‐beta peptide protects against microbial infection in mouse and worm models of Alzheimer's disease. Science Translational Medicine, 8, 340ra372. 10.1126/scitranslmed.aaf1059 PMC5505565 27225182

[acel12754-bib-0020] Li, W. L. , Cai, H. H. , Wang, B. , Chen, L. , Zhou, Q. G. , Luo, C. X. , … Zhu, D. Y. (2009). Chronic fluoxetine treatment improves ischemia‐induced spatial cognitive deficits through increasing hippocampal neurogenesis after stroke. Journal of Neuroscience Research, 87, 112–122. 18711744 10.1002/jnr.21829

[acel12754-bib-0021] Li, L. L. , Ginet, V. , Liu, X. , Vergun, O. , Tuittila, M. , Mathieu, M. , … Courtney, M. J. (2013). The nNOS‐p38MAPK pathway is mediated by NOS1AP during neuronal death. Journal of Neuroscience, 33, 8185–8201. 23658158 10.1523/JNEUROSCI.4578-12.2013 PMC6619617

[acel12754-bib-0022] Luo, C. X. , Lin, Y. H. , Qian, X. D. , Tang, Y. , Zhou, H. H. , Jin, X. , … Zhu, D. Y. (2014). Interaction of nNOS with PSD‐95 negatively controls regenerative repair after stroke. Journal of Neuroscience, 34, 13535–13548. 25274829 10.1523/JNEUROSCI.1305-14.2014 PMC6608318

[acel12754-bib-0023] Martel, M. A. , Ryan, T. J. , Bell, K. F. , Fowler, J. H. , McMahon, A. , Al‐Mubarak, B. , … Hardingham, G. E. (2012). The subtype of GluN2 C‐terminal domain determines the response to excitotoxic insults. Neuron, 74, 543–556. 22578505 10.1016/j.neuron.2012.03.021 PMC3398391

[acel12754-bib-0024] Misra, S. , Kuhad, A. , & Chopra, K. (2013). Neurobiological effect of 7‐nitroindazole, a neuronal nitric oxide synthase inhibitor, in experimental paradigm of Alzheimer's disease. Indian Journal of Experimental Biology, 51, 1086–1093. 24579374

[acel12754-bib-0025] Nikonenko, I. , Boda, B. , Steen, S. , Knott, G. , Welker, E. , & Muller, D. (2008). PSD‐95 promotes synaptogenesis and multiinnervated spine formation through nitric oxide signaling. Journal of Cell Biology, 183, 1115–1127. 19075115 10.1083/jcb.200805132 PMC2600742

[acel12754-bib-0026] Parsons, C. G. , & Rammes, G. (2017). Preclinical to phase II amyloid beta (Abeta) peptide modulators under investigation for Alzheimer's disease. Expert Opinion on Investigational Drugs, 26, 579–592. 28362514 10.1080/13543784.2017.1313832

[acel12754-bib-0027] Richier, L. , Williton, K. , Clattenburg, L. , Colwill, K. , O'Brien, M. , Tsang, C. , … Fawcett, J. P. (2010). NOS1AP associates with Scribble and regulates dendritic spine development. Journal of Neuroscience, 30, 4796–4805. 20357130 10.1523/JNEUROSCI.3726-09.2010 PMC5123869

[acel12754-bib-0028] Scheltens, P. , Blennow, K. , Breteler, M. M. , de Strooper, B. , Frisoni, G. B. , Salloway, S. , & Van der Flier, W. M. (2016). Alzheimer's disease. Lancet, 388, 505–517. 26921134 10.1016/S0140-6736(15)01124-1

[acel12754-bib-0029] Shen, L. , Yan, M. , & He, L. (2016). D5 receptor agonist 027075 promotes cognitive function recovery and neurogenesis in a Abeta1‐42‐induced mouse model. Neuropharmacology, 105, 72–83. 26773200 10.1016/j.neuropharm.2016.01.008

[acel12754-bib-0030] Soriano, F. X. , Martel, M. A. , Papadia, S. , Vaslin, A. , Baxter, P. , Rickman, C. , … Hardingham, G. E. (2008). Specific targeting of pro‐death NMDA receptor signals with differing reliance on the NR2B PDZ ligand. Journal of Neuroscience, 28, 10696–10710. 18923045 10.1523/JNEUROSCI.1207-08.2008 PMC2602846

[acel12754-bib-0031] Ul Islam, B. , Khan, M. S. , Jabir, N. R. , Kamal, M. A. , & Tabrez, S. (2017). Elucidating treatment of Alzheimer's Disease via different receptors. Current Topics in Medicinal Chemistry, 17, 1400–1407. 28049400 10.2174/1568026617666170103163715

[acel12754-bib-0032] Van Gelder, R. N. (2004). Resetting the clock: Dexras1 defines a path. Neuron, 43, 603–604. 15339641 10.1016/j.neuron.2004.08.029

[acel12754-bib-0033] Wilcox, K. C. , Lacor, P. N. , Pitt, J. , & Klein, W. L. (2011). Abeta oligomer‐induced synapse degeneration in Alzheimer's disease. Cellular and Molecular Neurobiology, 31, 939–948. 21538118 10.1007/s10571-011-9691-4 PMC3146579

[acel12754-bib-0034] Yu, S. Y. , Zhang, M. , Luo, J. , Zhang, L. , Shao, Y. , & Li, G. (2013). Curcumin ameliorates memory deficits via neuronal nitric oxide synthase in aged mice. Progress in Neuro‐Psychopharmacology and Biological Psychiatry, 45, 47–53. 23665290 10.1016/j.pnpbp.2013.05.001

[acel12754-bib-0035] Zadori, D. , Veres, G. , Szalardy, L. , Klivenyi, P. , Toldi, J. , & Vecsei, L. (2014). Glutamatergic dysfunctioning in Alzheimer's disease and related therapeutic targets. Journal of Alzheimer's Disease, 42(Suppl 3), S177–S187. 10.3233/JAD-132621 24670398

[acel12754-bib-0036] Zhang, J. , Huang, X. Y. , Ye, M. L. , Luo, C. X. , Wu, H. Y. , Hu, Y. , … Zhu, D. Y. (2010). Neuronal nitric oxide synthase alteration accounts for the role of 5‐HT1A receptor in modulating anxiety‐related behaviors. Journal of Neuroscience, 30, 2433–2441. 20164327 10.1523/JNEUROSCI.5880-09.2010 PMC6634557

[acel12754-bib-0037] Zhang, Y. , Li, P. , Feng, J. , & Wu, M. (2016). Dysfunction of NMDA receptors in Alzheimer's disease. Neurol Sci, 37, 1039–1047. 26971324 10.1007/s10072-016-2546-5 PMC4917574

[acel12754-bib-0038] Zhou, X. , Chen, Z. , Yun, W. , & Wang, H. (2015). NMDA receptor activity determines neuronal fate: location or number? Reviews in the Neurosciences, 26, 39–47. 25324444 10.1515/revneuro-2014-0053

[acel12754-bib-0039] Zhou, L. , Li, F. , Xu, H. B. , Luo, C. X. , Wu, H. Y. , Zhu, M. M. , … Zhu, D. Y. (2010). Treatment of cerebral ischemia by disrupting ischemia‐induced interaction of nNOS with PSD‐95. Nature Medicine., 16, 1439–1443. 10.1038/nm.2245 21102461

[acel12754-bib-0040] Zhou, L. , & Zhu, D. Y. (2009). Neuronal nitric oxide synthase: structure, subcellular localization, regulation, and clinical implications. Nitric Oxide, 20, 223–230. 19298861 10.1016/j.niox.2009.03.001

[acel12754-bib-0041] Zhu, L. J. , Li, T. Y. , Luo, C. X. , Jiang, N. , Chang, L. , Lin, Y. H. , … Zhu, D. Y. (2014). CAPON‐nNOS coupling can serve as a target for developing new anxiolytics. Nature Medicine, 20, 1050–1054. 10.1038/nm.3644 25129479

[acel12754-bib-0042] Zhu, L. , Tao, T. , Zhang, D. , Liu, X. , Ke, K. , & Shen, A. (2015). NOS1AP O‐GlcNAc modification involved in neuron apoptosis induced by excitotoxicity. International Journal of Molecular Sciences, 16, 16560–16575. 26197318 10.3390/ijms160716560 PMC4519966

