# Supplementary figures and images for: nNOS–CAPON interaction mediates amyloid‐β‐induced neurotoxicity, especially in the early stages
Source: Aging Cell. 2018 Mar 25;17(3):e12754. doi: 10.1111/acel.12754 (PMC5946066; doi:10.1111/acel.12754)

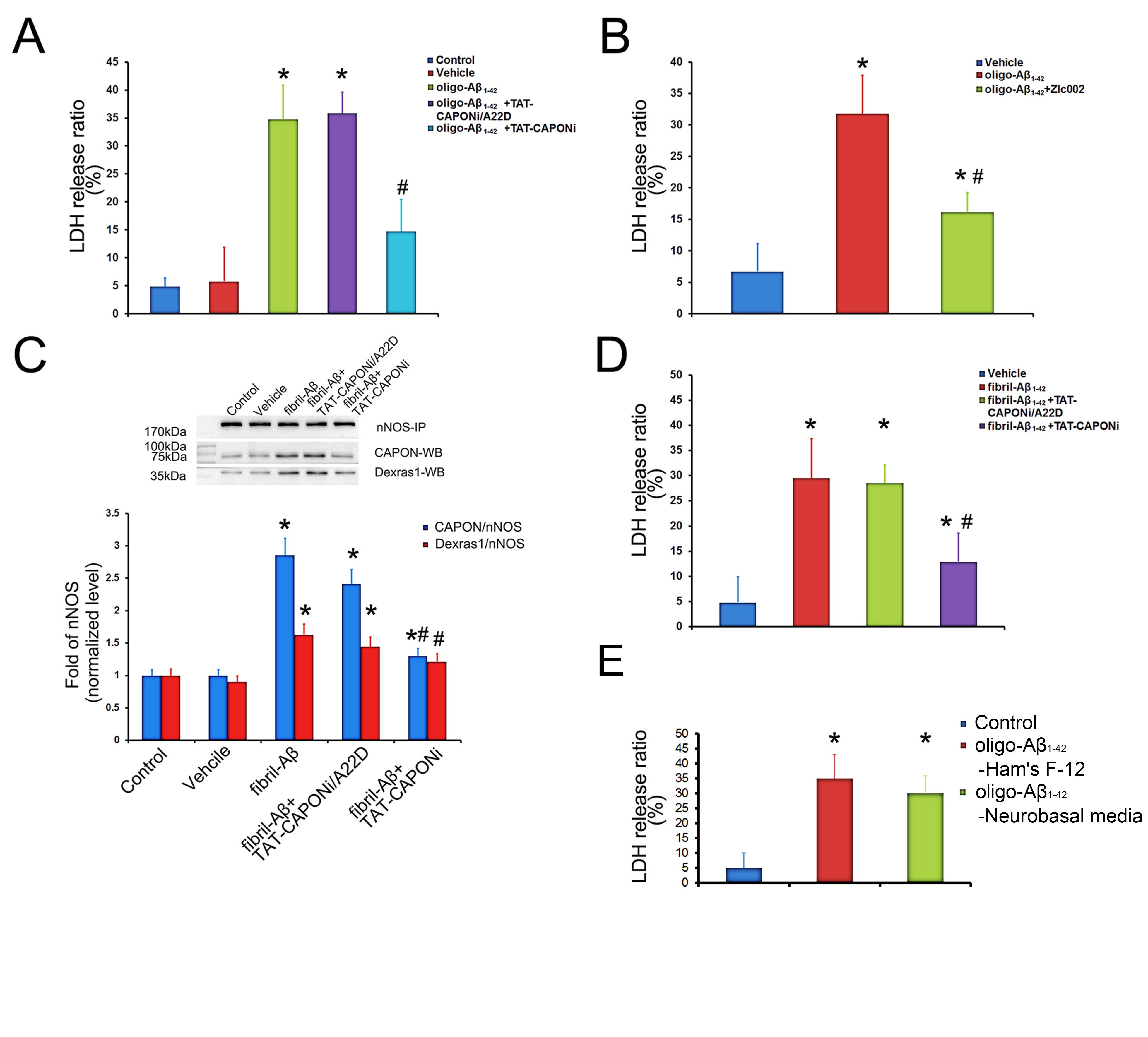

Supplement: Supplementary file 1 [file ACEL-17-e12754-s001.tif]

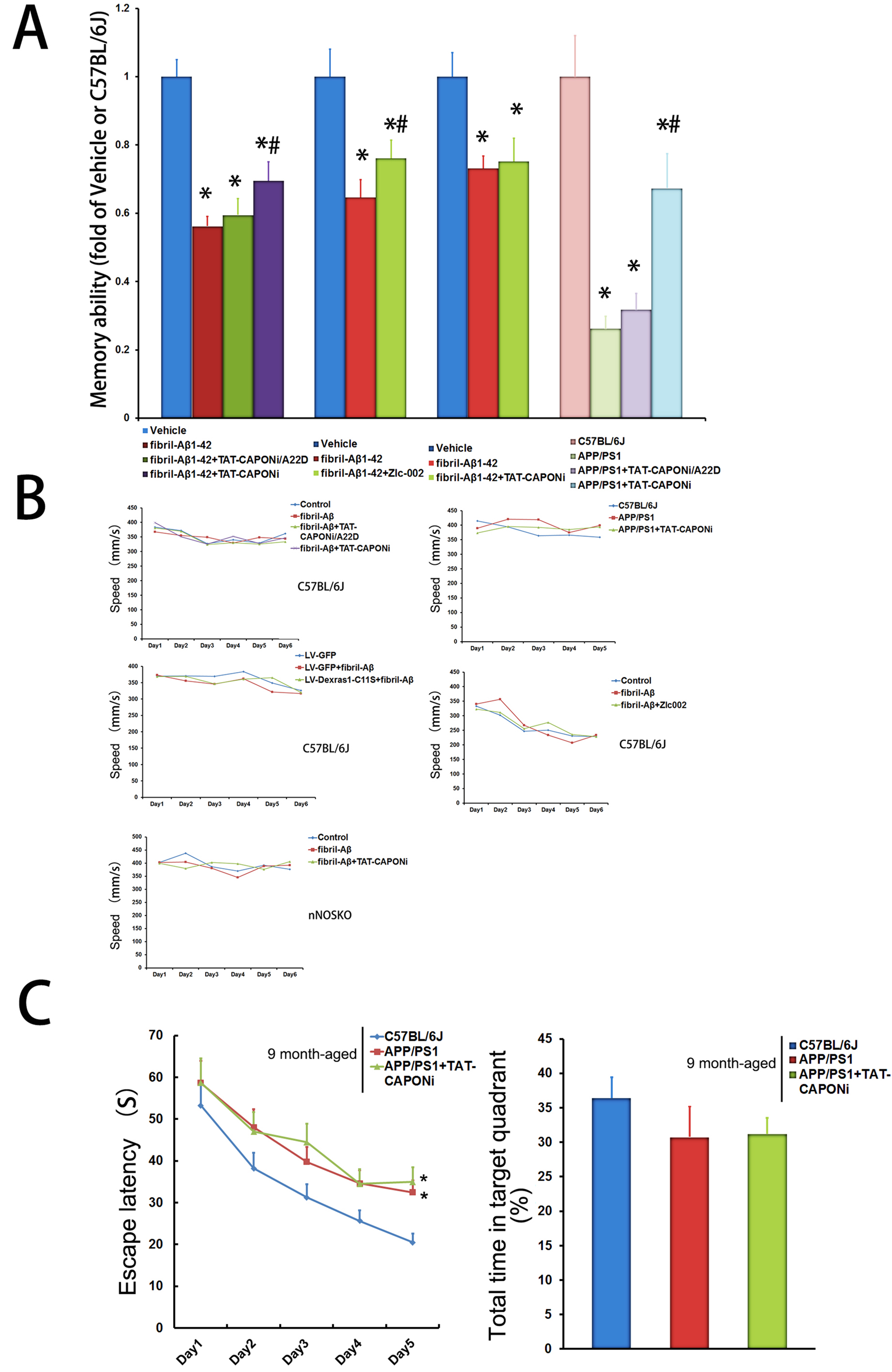

Supplement: Supplementary file 2 [file ACEL-17-e12754-s002.tif]

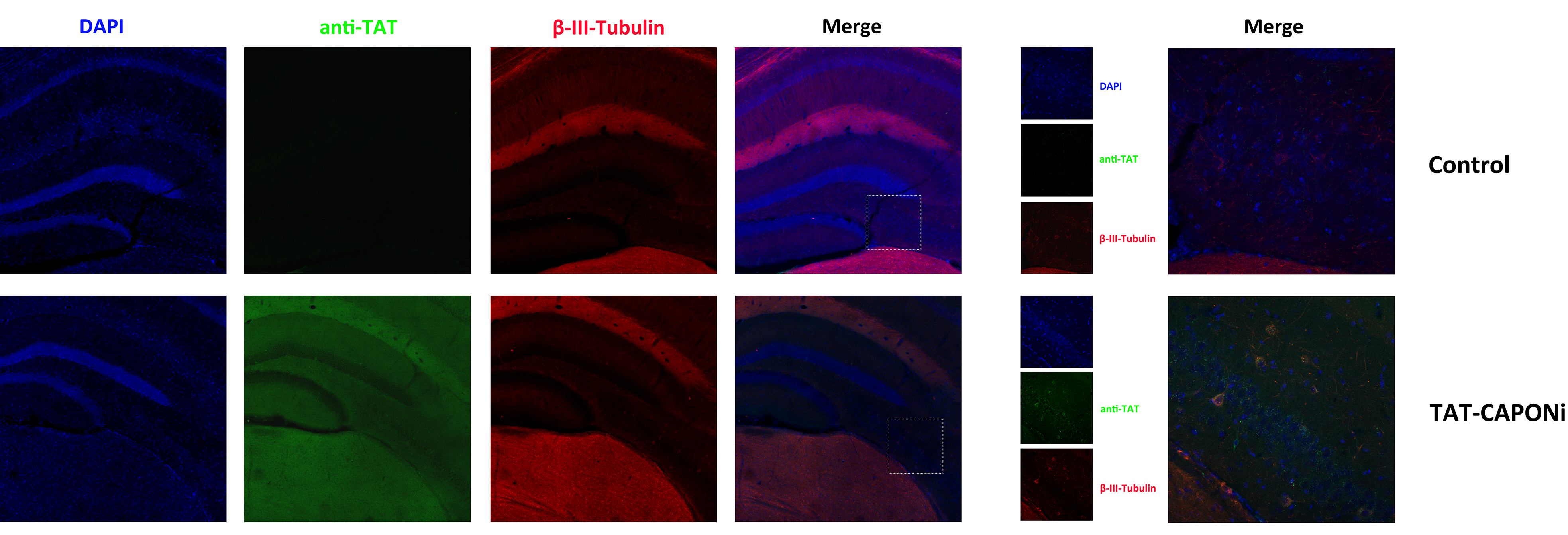

Supplement: Supplementary file 3 [file ACEL-17-e12754-s003.tif]

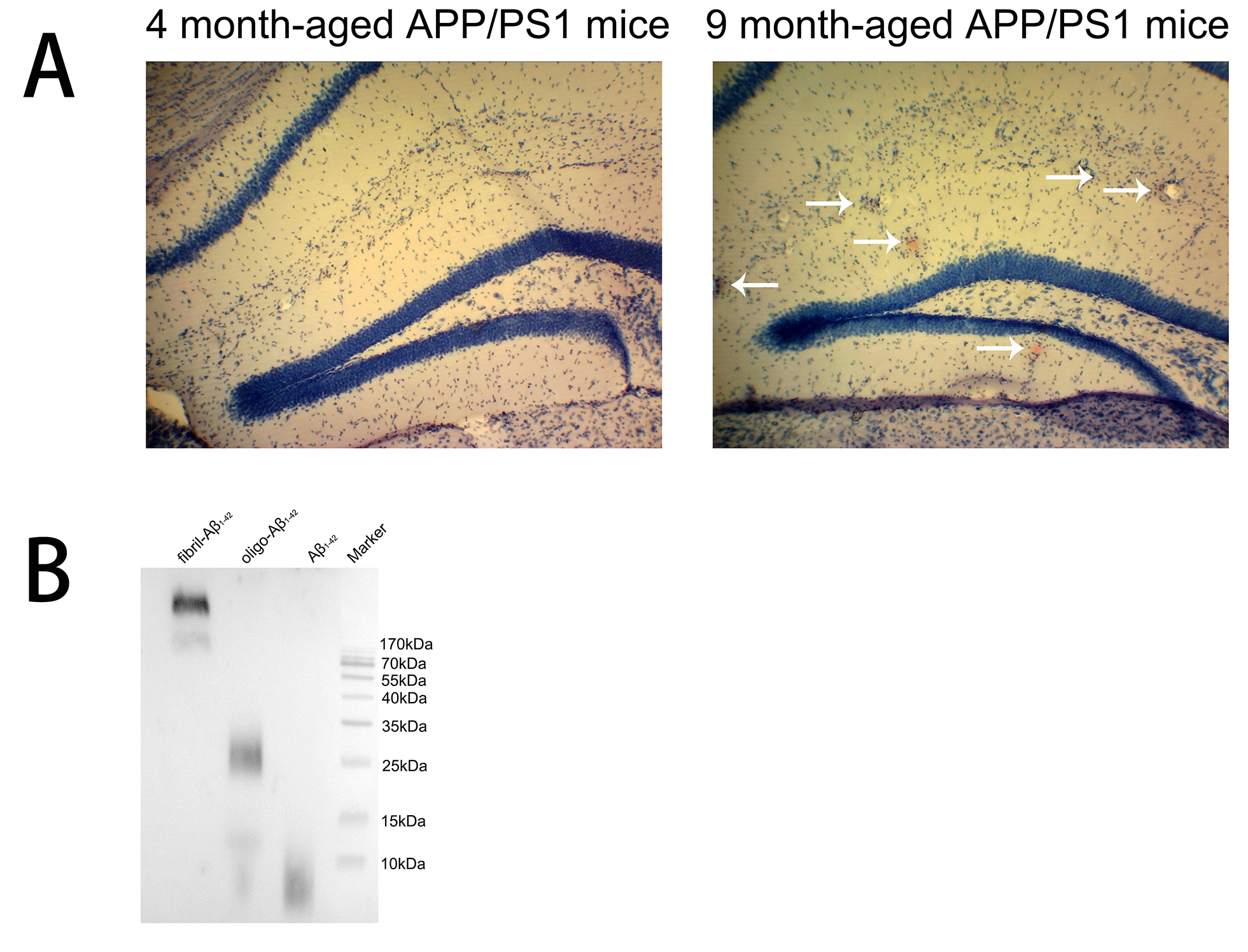

Supplement: Supplementary file 4 [file ACEL-17-e12754-s004.tif]
